# Neural mechanisms of emotion-modulated startle reflexes: insights from ERP and EEG oscillations

**DOI:** 10.7717/peerj.21136

**Published:** 2026-04-21

**Authors:** Fangfang Long, Lulu Hou, Renlai Zhou

**Affiliations:** 1 School of Psychology, Guizhou Normal University, Guiyang, Guizhou, China; 2 Department of Psychology, Nanjing Universtiy, Nanjing, Jiangsu, China; 3 School of Psychology, Shanghai Normal University, Shanghai, China; 4 Department of Radiology, Nanjing Drum Tower Hospital, Nanjing University, Nanjing, Jiangsu, China

**Keywords:** EMS paradigm, ERP analysis, ERO analysis, Startle reflex processing

## Abstract

**Background:**

The startle reflex is an involuntary response to a sudden, highly intense stimulus. Previous research has primarily focused on the early stages of the startle reflex, specifically examining the impact of emotional pictures on early responses such as the Probe P3 component. However, it remains unclear whether emotions affect the later stages of startle reflex processing. Furthermore, the influence of emotions on neural oscillations, such as theta and alpha waves, has not been sufficiently studied in this context.

**Methods:**

This study involved 27 participants (*N* = 27) and used emotional pictures (positive, negative, and neutral) together with a startle probe, combined with event-related potential (ERP) and event-related oscillation (ERO) analyses, to investigate the effects of emotions on both the early and later stages of the startle reflex.

**Results:**

The ERP results demonstrate that when applying the sudden sound stimuli, the negative picture condition evokes the smallest Probe P3 component and LPP, followed by the positive and neutral picture condition. More specifically, negative images reduced Probe P3 and LPP amplitudes compared with positive and neutral images, indicating sustained emotional modulation from early attentional to later processing stages. The ERO results show that early theta and alpha oscillations in the frontocentral region significantly differ between conditions, while the theta and alpha oscillations are largest when the sudden sound stimuli is applied in the negative picture condition, followed by the positive and neutral picture condition.

**Conclusion:**

These findings demonstrate that emotions exert sustained modulatory effects on both early and later stages of startle reflex processing, as reflected in ERP components and oscillatory neural activity. Together, the ERP and ERO results provide converging evidence for the neural dynamics underlying emotion-modulated startle responses.

## Introduction

The startle reflex is an involuntary response to a sudden, highly intense stimulus (Lang, Bradley & Cuthbert, 1997). It consists of a rapid continuous muscle contraction which occurs 30–50 ms after the onset of an auditory, dermatological or visual startle stimulus and then extends to the trunk and knees (Vrana, Spence & Lang, 1988; Blanch, Balada & Aluja, 2013). While these physiological characteristics are well established, recent research has increasingly focused on how emotional states modulate neural responses to startle probes within the emotion-modulated startle (EMS) paradigm.

In humans, the startle reflex in response to a startle probe is the most easily measured and stable form of this reflex. Typically, this involves the presentation of white noise or a pure tone at 90–110 dB through headphones for 20–500 ms. With the development of sound-startle techniques, researchers introduced the EMS paradigm to investigate how emotional contexts influence the strength of the startle reflex (Blanch, Balada & Aluja, 2013; Landis & Hunt, 1939). A common manipulation consists of applying a sudden sound startle stimulus during picture presentation and observing the participants’ startle reflex to different emotional pictures. Previous studies have shown that reflexive responses to sudden sound startle stimuli are modulated by emotional pictures. For instance, in the study by Lang (1995), participants were given a startle probe during picture viewing, and the blink response measured by electromyography (EMG) was used as an indicator of the startle reflex. Results indicated that the intensity of the startle reflex was higher when viewing negative pictures compared to neutral pictures, while the reflex intensity decreased when viewing positive pictures, particularly those with high arousal.

Similar results were obtained in studies using electroencephalography (EEG) to measure the startle reflex. In higher temporal resolution EEG measurements, a commonly used ERP indicator is the Probe P3 component (or Probe P300) produced by participants in response to sudden secondary sounds when viewing emotional pictures, peaking around 300 ms (Bradley, Codispoti & Lang, 2006; Cuthbert et al., 2000). Probe P3 reflects the amount of resources allocated to sound stimuli during emotional picture processing (Bradley, Codispoti & Lang, 2006). Specifically, Probe P3 amplitude decreases when participants view emotional pictures compared to neutral ones, suggesting that emotional pictures capture more attention, leaving fewer resources for processing sound stimuli (Schupp et al., 1997). Moreover, it has been shown that Probe P3 is affected by emotional arousal, with its amplitude being significantly smaller only when sound stimuli are applied during the presentation of highly arousing emotional pictures (Leite et al., 2012).

Importantly, prior EEG studies have consistently demonstrated that emotional contexts modulate startle-probe processing. Probe P3 suppression has been widely reported when the probe is delivered during emotionally salient picture viewing, reflecting prioritized emotional stimulus processing over the startle probe (Bradley, Codispoti & Lang, 2006; Keil et al., 2007). In addition to early Probe P3 effects, research on emotional picture viewing has shown that emotional influences continue into later stages, indexed by sustained late positive potential (LPP) activity (Hajcak & Olvet, 2008). However, relatively few studies have examined whether such late-stage emotional modulation extends to startle-evoked neural responses, indicating that additional clarification in this area remains valuable.

Beyond ERP analysis, event-related oscillations (ERO) analysis offers insights into non-time-locked and non-phase-locked components in EEG signals. Neural oscillations in different frequency bands are associated with various cognitive activities and are modulated by emotional factors (Başar et al., 2000). For example, Uusberg et al. (2013) found that emotional content increases alpha oscillations, while Lesting et al. (2011) reported that theta oscillatory activity increases in a negative fear context, distinguishing it from neutral and positive contexts. These studies suggest that alpha and theta oscillations can reflect differences in processing activity under different emotional influences. Moreover, substantial work using IAPS emotional pictures has demonstrated that oscillatory responses systematically vary with valence and arousal, such that negative or high-arousal images induce stronger theta event-related synchronization and more pronounced alpha suppression (Aftanas et al., 2001; Luther et al., 2023). In addition, evidence from acoustic startle paradigms indicates that startle-related neural oscillations are functionally linked to defensive processing, as prepulse inhibition of the startle reflex is accompanied by parallel inhibition of auditory-evoked theta oscillations (Kedzior, Koch & Basar-Eroglu, 2006), suggesting that low-frequency oscillations can index startle-related sensory gating and emotional reactivity. However, oscillatory responses specifically time-locked to startle probes under emotional conditions have received limited investigation (Güntekin & Başar, 2014), and it remains unclear whether theta and alpha power can serve as sensitive neural markers of emotion-modulated startle processing.

Taken together, these findings highlight the value of integrating EEG-based measures into emotion-modulated startle paradigms. By capturing both time-locked (ERP) and non-time-locked (ERO) neural dynamics, EEG provides a more comprehensive perspective on the neural mechanisms through which emotional contexts shape defensive reflexes. Compared with traditional startle paradigms that rely primarily on peripheral physiological indices, EEG-based approaches allow for a more direct examination of how emotional significance influences attentional allocation, sensory gating, and sustained defensive processing at the neural level.

Moreover, converging evidence from ERP and oscillatory studies suggests that emotion-modulated neural responses within the EMS paradigm reflect systematic variations in emotional and attentional processing, particularly under conditions involving motivationally salient or threat-related stimuli. Components such as Probe P3 and the late positive potential (LPP), together with low-frequency oscillatory activity, have been shown to index prioritized emotional processing and the allocation of attentional resources (Bradley, Codispoti & Lang, 2006; Cuthbert et al., 2000; Hajcak & Olvet, 2008; Güntekin & Başar, 2014). As such, EMS-derived EEG markers offer a valuable window into the dynamic interaction between emotional significance and defensive responding, beyond what can be inferred from reflex magnitude alone. However, despite growing evidence linking ERP components and oscillatory activity to emotion-modulated defensive processing, it remains unclear how emotional factors jointly shape both early and later stages of startle-evoked neural responses across time and frequency domains.

This study aims to examine how emotional factors modulate both early and later stages of startle reflex processing at the neural level. Using time-domain analyses, we investigate whether emotional influences extend beyond early attentional stages into later processing phases, as reflected by differences in the Probe P3 and LPP components when startle sounds are applied under different emotional picture conditions. In the frequency domain, we further examine whether emotional factors selectively modulate lower-frequency oscillatory activity (theta and alpha), which has been implicated in emotional and defensive processing. To induce different emotional conditions, we use high arousal negative pictures, high arousal positive pictures, and neutral pictures, following previous studies. To ensure a robust startle effect, sound startle stimuli are applied to only half of the pictures. Two independent variables are manipulated in this study: picture type and the presence or absence of sound stimuli. Previous studies have shown that the sudden startle sound should be presented midway through the picture presentation to ensure that the startle reflex is influenced by the emotional content (Bradley, Codispoti & Lang, 2006; Leite et al., 2012). If the sound stimulus is presented too soon after the picture appears, such as at 150 or 300 ms, the startle reflex may only be influenced by sensory gating rather than emotional content. Therefore, in this study, the startle stimulus is applied halfway through the picture presentation.

## Materials and Methods

### Participants

Thirty college students enrolled in Guizhou Normal University were recruited for this experiment. The sample size required for the experiment was calculated using the G*POWER 3.1 software (Faul et al., 2007). Referring to a previous study on event-related potentials for startle reflexes and the criteria suggested by Cohen (1992), the parameters were set as follows: F test, ANOVA-repeated measure, within factors, effect size = 0.25, α = 0.05, 1 − β = 0.8, number of groups = 1 and number of measurements = 6. This program was then used to obtain a “total sample size” of 19. However, considering that some of the noisy sample data may be removed, a sample size of 30 subjects was chosen. Three participants were excluded from data analysis because more than 50% of their EEG epochs were contaminated by artifacts, resulting in poor overall data quality. Therefore, a total of 27 participants’ data (14 females, mean age ± *SD* = 23.31 ± 1.17; 13 males, mean age ± *SD* = 23.12 ± 1.92) were used for statistical analysis. All the participants were right-handed, had no history of mental illness, and had normal hearing and vision. This study was approved by the Ethics Committee of Guizhou Normal University (Approval number: GZNUPSY.N.202309E [0019]). In addition, all the participants signed an informed consent form before the experiment and received a cash of 60 Yuan after the experiment.

Following recent evaluations of sample-size calculations performed with G*Power (Thibault et al., 2024), the initially adopted effect size (*f* = 0.25) corresponds to a conventional “medium” effect size rather than an empirically derived estimate. Under more appropriate assumptions for a 3 × 2 within-subject repeated-measures design, an effect size of this magnitude corresponds to a partial $\eta^2$ of approximately 0.059 and would require a larger sample of about 44 participants to achieve 80% power. Therefore, the current sample of 27 participants should be considered relatively underpowered for detecting small effects, and the findings should be interpreted with caution regarding statistical power.

### Materials

#### Picture materials

Eighty negative images (560 × 420 pixels), eighty positive images (560 × 420 pixels) and eighty neutral images (560 × 420 pixels) from the International Library of Emotional Images (Lang, Bradley & Cuthbert, 1997) were used as emotional stimulus material. Each selected picture showed high consistency in emotion classification. The pictures were selected based on Chinese normative ratings of the International Affective Picture System (IAPS), as established by Liu, Xu & Zhou (2009), which provides localized valence and arousal norms for Chinese university students. The valence score of all the negative pictures was 2.28 ± 1.54, and the corresponding arousal score was 5.84 ± 2.49. The valence score of all the positive pictures was 7.02 ± 1.65, and the corresponding arousal score was 5.84 ± 2.04. The valence score of all the neutral pictures was 4.95 ± 1.48, and the corresponding arousal score was 4.25 ± 1.85. The analysis of variance (ANOVA) results showed that the three types of pictures were significantly different in valence (*F* (2,158) = 1,463.974, *p* < 0.001) and arousal (*F* (2,158) = 356.276, *p* = 0.001). To ensure that both emotional pictures had high arousal, the negative and positive pictures were selected with high arousal. The *post hoc* analysis showed no significant difference in arousal between the negative and positive pictures (*p* = 0.984), but both negative and positive pictures had significantly higher arousal than neutral pictures (negative *vs* neutral: *p* < 0.001; positive *vs* neutral: *p* < 0.001). The number of pictures was provided in Supplemental Material (Part 1).

#### Sudden sound startle stimulus

For the abrupt sound startle stimulus, a white noise was generated using the Psychophysics Toolbox (PTB) program, and then adjusted to 105 dB in the ear. During the experiment, the central fixation (0.2°) before picture presentation was set to dithering presentation between 800 and 1,000 ms to avoid participants’ anticipation of the picture stimuli.

### Procedure

**Figure 1:**
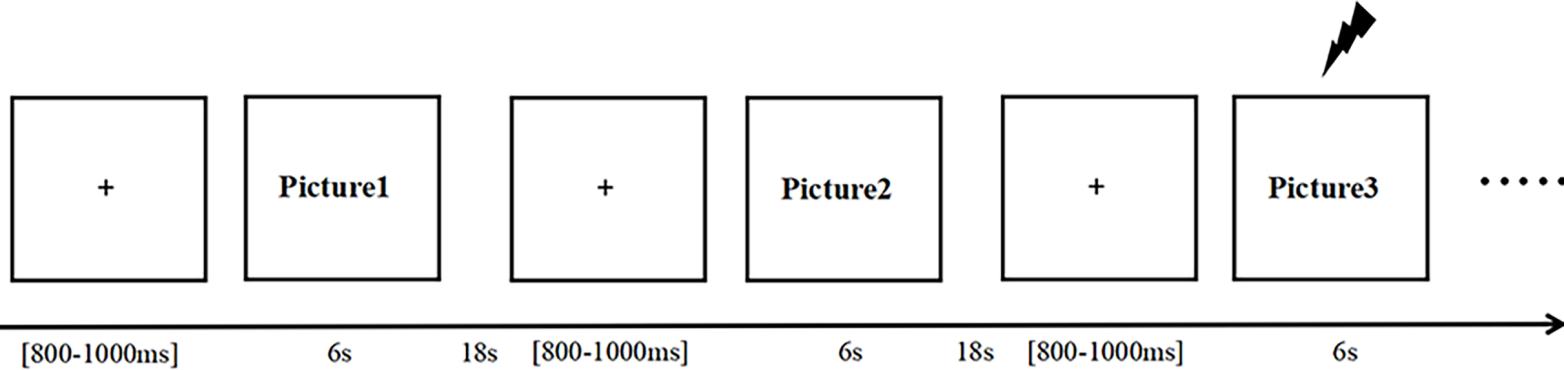
Experimental flowchart.

The experiments were performed in a quiet and comfortable room with soft light and good electromagnetic shielding. The experimental procedure was presented by the PTB software with a computer monitor placed 65 cm in front of the participant’s line of sight. The auditory stimuli were presented binaurally by Beats headphones at a volume level of 105 dB in the ear. The experimental procedure is shown in Fig. 1.

In this figure, the lightning symbol represents the startle probe (a 105 dB white noise), and each trial began with a fixation cross displayed at the center of the screen.

In this study, three different types of picture stimuli (negative, positive and neutral) were randomly presented. The experiment consisted of eight block tasks, each with 30 trials, for a total of 240 trials. In each trial, after the presentation of the fixation cross, pictures were presented for 6 s. In half of the trials, a white noise of 105 dB (startle probe) was randomly presented at the 4th second after the onset of the picture stimulus, lasting for 50 ms. The same number of trials with sound stimuli appeared for each type of picture. After the pictures were presented, there was an 18-s interval to allow participants to return to baseline levels, and after the 18-s interval, the next trial started.

### EEG recording

EEG data were recorded from 64 Ag/AgCl electrodes mounted in an elastic cap according to the modified 10–20 International system (ANT Neuro EEGO Inc., Berlin, Germany). The continuous EEG data were recorded with a 100 Hz low-pass filter with a sampling rate of 500 Hz. The recordings were initially referenced to CPz. The impedances were kept below 5 KΩ for all the electrodes.

After the acquisition was completed, the data were preprocessed using EEGLAB (Delorme & Makeig, 2004). More precisely, the data were first re-referenced using bilateral mastoids as reference electrodes. They were then low-pass filtered at 0.1 Hz and high-pass filtered at 30 Hz. Afterwards, the bad electrode data were replaced using interpolation, and the bad segments were rejected. In addition, other artifacts with amplitudes greater than ±80 μV were automatically excluded. Finally, eye movement artifacts were corrected using individual independent component analysis (ICA) by removing the corresponding components based on the particular activation curve (Mennes et al., 2010).

### Data processing and analysis

#### ERP analysis

To characterize neural responses elicited by picture presentation prior to the startle probe, participants’ EEG responses during picture viewing were first analyzed. We called it passive viewing of the pictures. In the fourth second of the picture appearance, the operation of applying/not applying the startle probe was started. At this stage, we examined neural responses not only in the three emotional picture conditions with the sound stimulus, but also in the corresponding conditions without the sound stimulus. By analyzing the three picture conditions in the presence or absence of sound, it was possible to clarify that the participants’ responses at this point were primarily driven by the sound stimulus rather than by picture presentation alone.

In the passive viewing of the pictures, ERP analysis intercepted the time period from 200 ms before the image was presented (as a baseline) to 1,000 ms after the image was presented. During the statistical analysis, the within-subject factor was the picture type (positive, neutral, negative). The P3 time window was defined as 300–400 ms. This selection was primarily based on the peak latency and scalp distribution observed in the present study’s waveforms and topographic maps. Importantly, this range falls within the broader 300–600 ms interval commonly associated with posterior P3 activity during emotional picture perception (Schupp et al., 2000). Similarly, the LPP window was defined as 400–900 ms, consistent with both the sustained parietal positivity in our dataset and prior literature showing that the LPP typically begins around 400 ms and can persist for several hundred milliseconds (Hajcak & Olvet, 2008). In combination with the topographic maps of this study, the average of 10 electrodes in the occipital region (PZ, POz, P5, P3, PO5, PO3, P6, P4, PO4 and PO6) was therefore used for P3 (300–400 ms), and the average of nine electrodes in the parietal region (CP1, CP2, CPz, P1, P2, Pz, PO3, PO4, and POz) was used for LPP (400–900 ms).

For the startle probe analysis, EEG data were segmented using a time window of [−200, 1,000] ms centered around the onset of the startle probe. Therefore, the six conditions were: negative picture + with sound, positive picture + with sound, neutral picture + with sound, negative picture + without sound, positive picture + without sound and neutral picture + without sound. In this section, the within-subject factors include the picture type (positive, neutral, negative) and sound (with sound, without sound). Probe P3 and LPP components were considered for statistical analysis. Inspection of the Probe P3 waveforms showed that although the positivity began to decline after approximately 350 ms, the peak consistently occurred within the 300–400 ms interval across conditions, and the descending flank of the component remained above baseline until close to 400 ms. Therefore, selecting a 300–400 ms window allowed us to capture the full probe-evoked positivity while avoiding later activity unrelated to the P3. This window also falls within the broader 300–600 ms range commonly used for startle-probe P3 components during emotional picture viewing (Schupp et al., 1997; Bradley, Codispoti & Lang, 2006). The electrodes analyzed for Probe P3 were the average amplitudes of electrodes in the frontocentral region (F1, F2, F3, F4, Fz, FC1, FC2, FC3, FC4, and FCz), consistent with prior findings indicating that probe-elicited P3 activity typically shows a frontocentral maximum (Keil et al., 2007), reflecting attentional allocation processes rather than posterior positivity. For the LPP in this section, the 400–900 ms time window was again adopted, consistent with both our topographic distribution and standard LPP definitions in affective ERP research (Hajcak & Olvet, 2008). The electrodes used were CP1, P1, CPZ, PZ, POZ, CP2 and P2.

All the data analyses were performed using SPSS 22.0, and ANOVA spherical test failure was corrected using the Greenhouse-Geisser method, with significance level using *p* < 0.05 and effect size expressed as *ηp*^2^.

#### ERO analysis

The ERO analysis was performed using the short-time Fourier transform method with a Hanning window having a width of 200 ms. For each trial, one short-time Fourier transform is performed at each time point and each frequency band. The resulting spectrogram, representing the joint function of time and frequency of signal power at each time-frequency point, that is $\rm {P (t, f) = \Vert f(t, f)\Vert^2}$, includes the brain’ s response to phase locking (event-related potentials) and non-phase locking (event-related synchronization and desynchronization) (Hu et al., 2014; Mouraux & Iannetti, 2008). The time window from 500 ms to 200 ms before stimulus onset was used as the baseline for correction. Because time–frequency analysis requires a longer temporal window to obtain stable oscillatory estimates and reduce edge effects, the ERO time window for probe-related theta/alpha activity (0–400 ms) was intentionally extended beyond the corresponding ERP latency range, whereas the 400–900 ms window for LPP-related oscillations followed the conventional time range used in affective ERP research. For the ERO analysis, this study focuses only on the neural oscillatory response after the presence or absence of the sound stimulus. Therefore, the data were analyzed for the six conditions of the presence or absence of sound after the fourth second. In this section, statistical analyses were performed for the theta frequency band (4–7 Hz) and alpha frequency band (8–12 Hz) in the frontocentral and occipital regions, respectively, with reference to the regions where differences existed at the time of ERP analysis. All the data analyses were performed using the SPSS 22.0 software, and the failure of the repeated measures ANOVA sphericity test was corrected using the Greenhouse-Geisser method, while those involving multiple comparisons were corrected using Bonferroni. The significance levels were *p* < 0.05, and the effect sizes were expressed as *ηp*^2^.

## Results

### ERP results for passively viewed images

**Figure 2:**
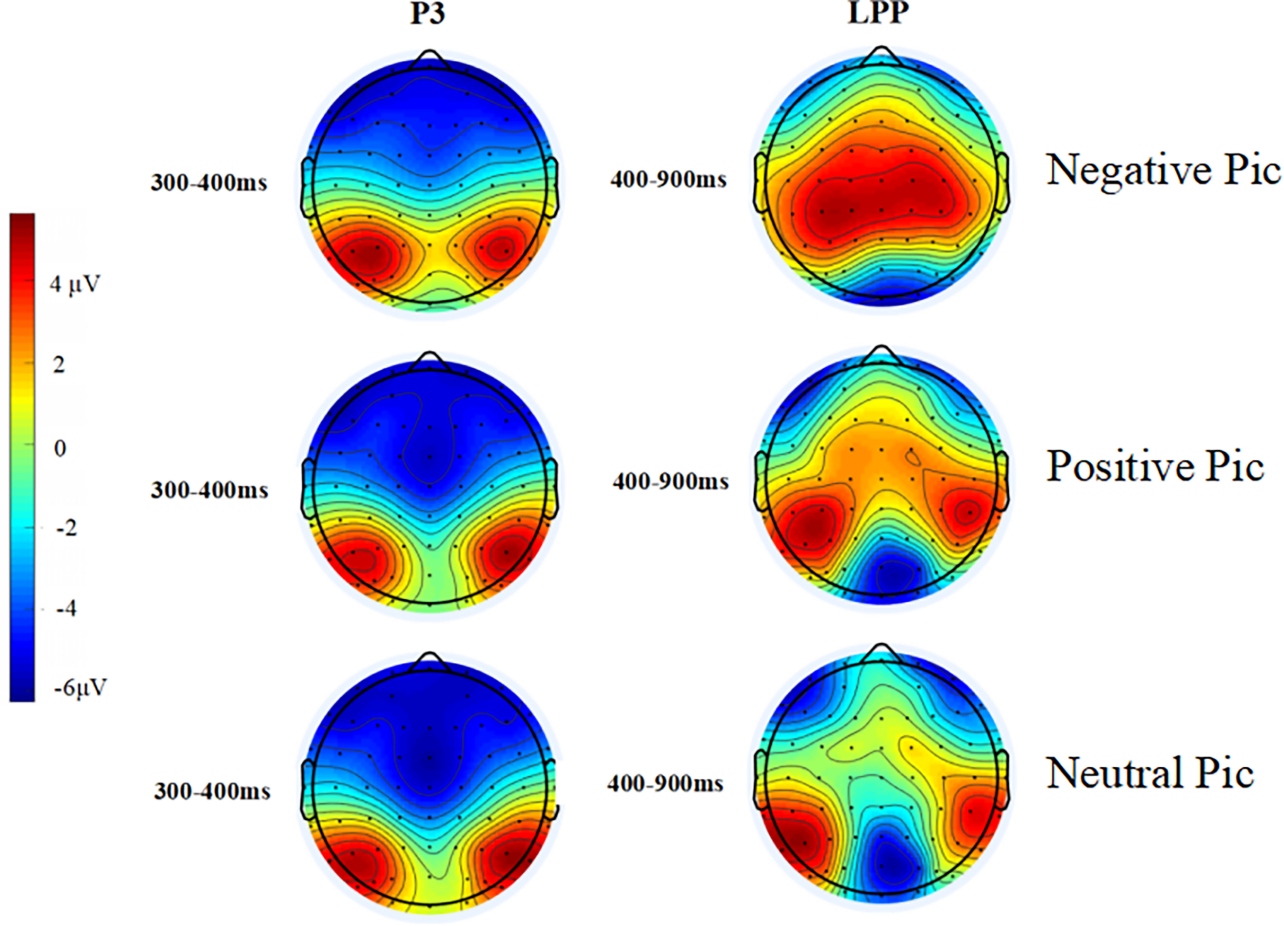
Passive viewing of the topographic map of the picture.

According to the topography of the participants (Fig. 2), it is clear that the activation of P3 is mainly in the occipital region, while LPP is mainly in the parietal region. Therefore, the amplitude of P3 is the average wave amplitude of the electrodes in the occipital region during the time window of 300–400 ms, and LPP is the average wave amplitude of the electrodes in the parietal region during the time window of 400–900 ms.

**P3**

A one-way (picture type: negative, positive, neutral) repeated measures ANOVA is performed for the P3 amplitude. The results violate the sphericity assumption, and they are corrected using the Greenhouse-Geisser method.

**Figure 3:**
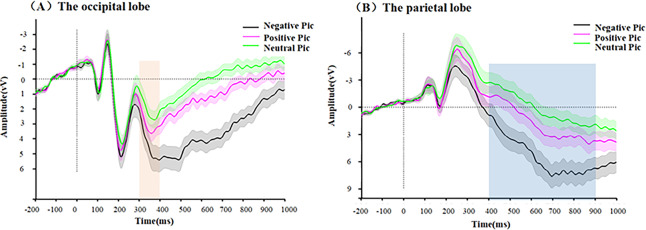
Waveform plots during passive viewing of the images. (A) The waveforms in the occipital region, (B) the waveforms in the parietal region.

The results indicate a significant main effect of condition (*F* (1.4,36.9) = 17.91, *p* < 0.001, *ηp*^2^ = 0.408). A pairwise comparison analysis showed that the differences between all three conditions were significant: negative *vs* positive (*p* = 0.004), negative *vs* neutral (*p* < 0.001), and positive *vs* neutral (*p* < 0.001). The negative picture condition has the largest amplitude of P3 (*M* = 4.43 ± 4.11), followed by the positive picture condition (*M* = 3.05 ± 2.58) and finally the neutral picture condition (*M* = 2.10 ± 2.92). All descriptive data are reported as mean ± standard deviation (SD). The waveform diagram of the P3 component is shown in Fig. 3A.

**LPP**

A one-way (picture type: negative, positive, neutral) repeated measures ANOVA is performed on the LPP amplitude. The results violate the sphericity assumption condition, and they are reported in this section after correction using the Greenhouse-Geisser method.

The results show a significant main effect of condition (*F* (1.3,33.9) = 58.10, *p* < 0.001, *ηp*^2^ = 0.691). A pairwise comparison analysis shows that the differences between all the three conditions are significant. The largest magnitude of LPP is found in the negative picture condition (*M* = 5.42 ± 6.26), followed by the positive picture condition (*M* = 1.84 ± 5.31) and finally the neutral picture condition (*M* = 0.16 ± 4.90). The waveform diagram of the LPP component is shown in Fig. 3B.

### ERP and ERO result after applying/not applying acoustic startle stimuli

#### ERP result

According to the topography of the participants, the activation of the Probe P3 component in each condition is mainly in the frontocentral region, while the activation of the LPP is mainly in the occipital region. Therefore, the wave amplitude of Probe P3 is the average amplitude of electrodes in the frontocentral region at 300–400 ms, and LPP is the average amplitude of electrodes in the occipital region at 400–900 ms. Additional scalp topographies for the trials without acoustic startle stimuli are provided in Supplemental Material (Part 2).

**Probe P3**

**Figure 4:**
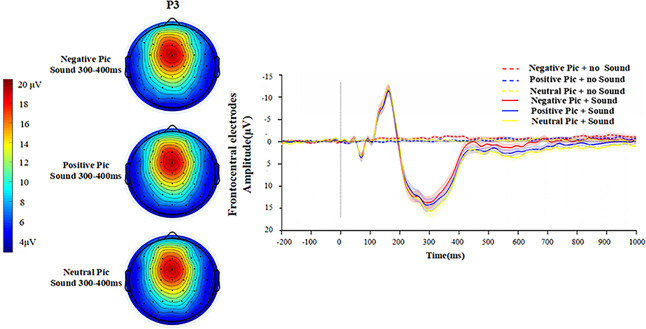
Waveforms in prefrontal conditions after the appearance of sound stimuli. The topographic map on the left presents only the topographic maps of the P3 components in the three emotional picture conditions with the presence of sound stimuli.

A 2 (with/without sound) × 3 (picture type: negative, positive, neutral) repeated measures ANOVA is performed on the Probe P3 amplitude. The results in Fig. 4 show that the main effect of sound condition (*F* (1,26) = 64.412, *p* < 0.001, *ηp*^2^ = 0.712), the main effect of emotion picture type (*F* (2,52) = 8.305, *p* = 0.001, *ηp*^2^ = 0.242) and the interaction between the two factors (*F* (2,52) = 3.679, *p* = 0.032, *ηp*^2^ = 0.124), are significant.

A simple effects analysis reveals significant differences in Probe P3 wave amplitude between the three emotion picture conditions in the presence of sound stimuli (*F* (2,52) = 9.082, *p* < 0.001, *ηp*^2^ = 0.259). More precisely, significant differences in wave amplitude between the negative picture condition and the positive picture condition (*p* = 0.027), the negative picture condition and the neutral picture condition (*p* < 0.001), the positive picture condition and the neutral picture condition, is not significant (*p* = 0.057). The largest Probe P3 wave amplitude is found in the neutral picture condition (*M* = 10.42 ± 6.12), followed by the positive picture condition (*M* = 9.43 ± 6.48) and finally the negative picture condition (*M* = 8.31 ± 6.68). On the contrary, there is no significant difference in Probe P3 wave amplitude in the three emotional picture conditions when no sound stimuli are present (*F* (2,52) = 0.355, *p* = 0.703, *ηp*^2^ = 0.013).

**LPP**

**Figure 5:**
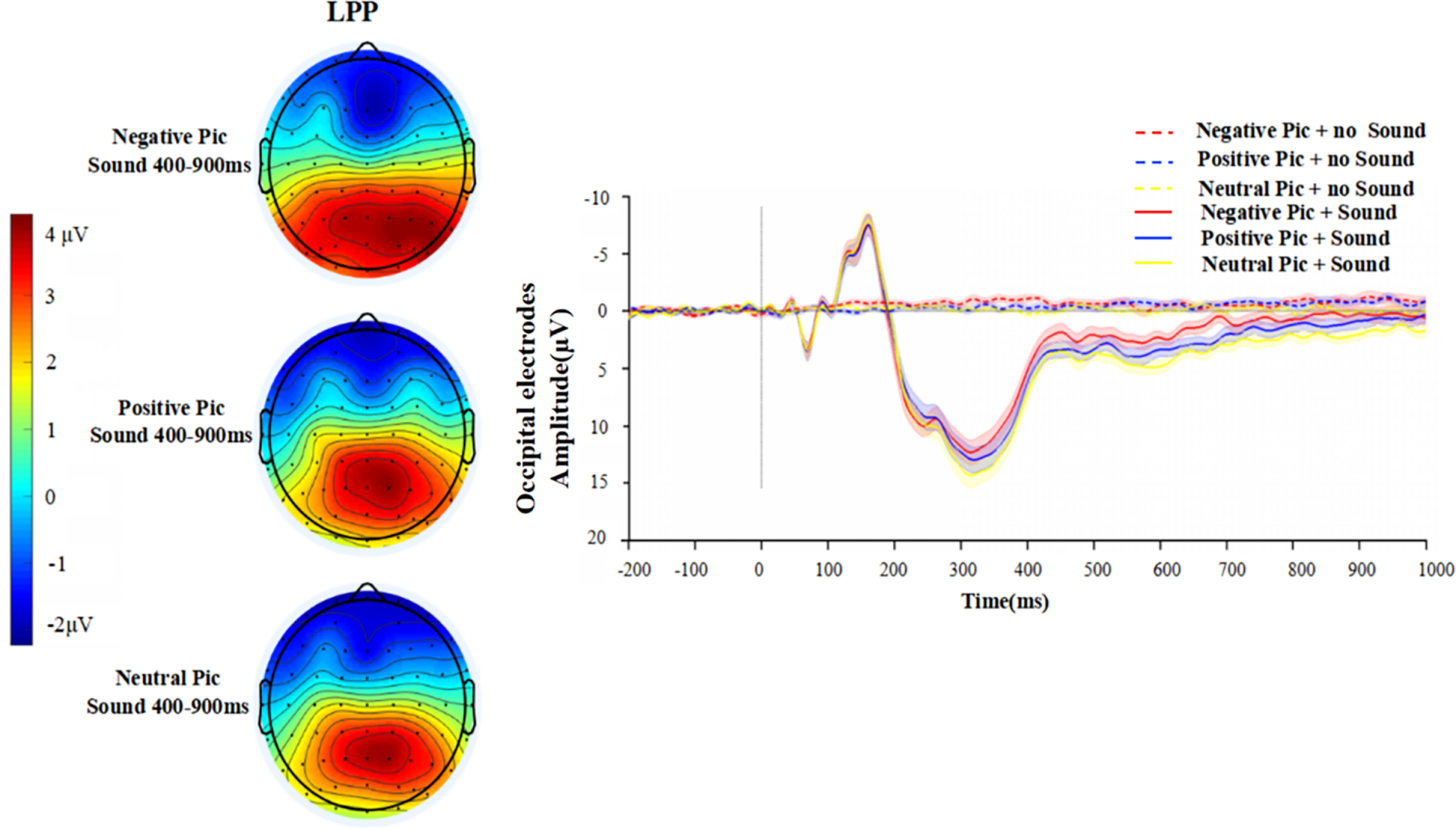
Waveforms in the occipital region in each condition after the appearance of sound stimuli. The topography on the left presents only the topography of LPP components in the three mood picture conditions with the presence of sound stimuli.

A 2 (with/without sound) × 3 (picture type: negative, positive, neutral) repeated measures ANOVA is performed on the LPP amplitude. The results in Fig. 5 show that the main effect of sound condition (*F* (1,26) = 46.18, *p* < 0.001, *ηp*^2^ = 0.640) and the main effect of emotion picture type (*F* (2,52) =11.56, *p* < 0.001, *ηp*^2^ = 0.308) are significant, while the interaction of the two factors is not significant (*F* (2,52) = 2.50, *p* = 0.092, *ηp*^2^ = 0.088).

A simple effects analysis shows that there is a significant difference in LPP amplitude between the three emotion picture conditions when there is a sound startle stimulus present (*F* (2,52) = 11.31, *p* < 0.001, *ηp*^2^ = 0.303). More precisely, the difference in wave amplitude between the negative picture condition and the positive picture condition is significant (*p* = 0.014), the difference in amplitude between the negative picture condition and the neutral picture condition is significant (*p* < 0.001), and the difference between the positive picture condition and the neutral picture is not significant (*p* = 0.051). The neutral picture condition has the largest LPP amplitude (*M* = 3.43 ± 2.52), followed by the positive picture condition (*M* = 2.62 ± 2.63) and the negative picture condition (*M* =1.57 ± 2.49). However, there is no significant difference in LPP wave amplitude between the three emotion picture conditions when no sound startle stimulus is applied (*F* (2,52) = 2.05, *p* = 0.139, *ηp*^2^ = 0.034).

#### The ERO result

**Figure 6:**
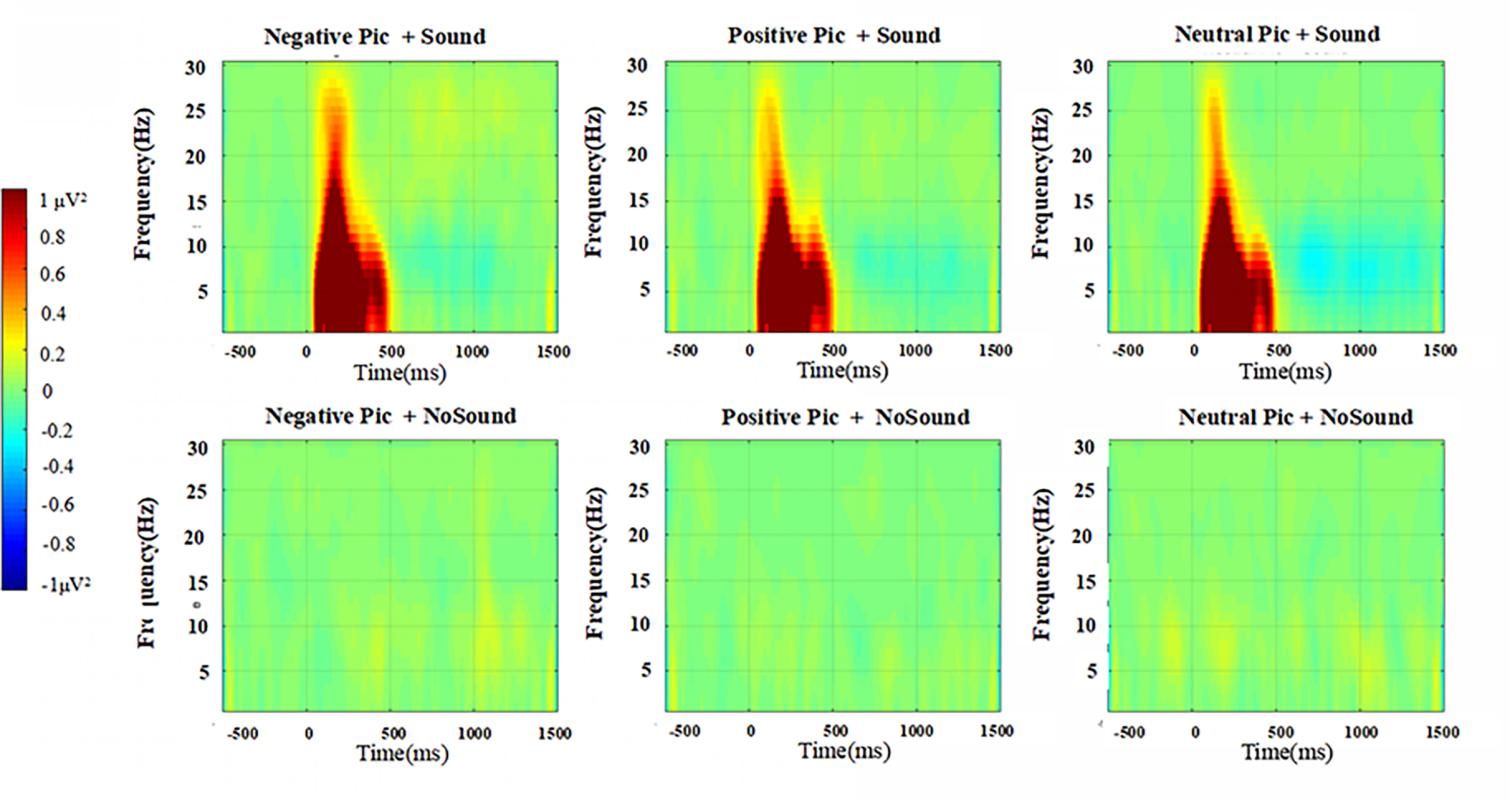
Time-frequency maps of prefrontal regions with/without startle stimuli.

ERO analysis of the frontocentral regions is performed, where the Probe P3 component appears in the ERP analysis as well as the occipital regions where the LPP component appears. The main focus is on the low frequency theta band (4–7 Hz) and the alpha band (8–12 Hz). For the selection of time windows, considering that the time-frequency analysis requires a longer window to obtain a stable result, this study does not directly use the time window of Probe P3 (300–400) for the analysis in the frontocentral region, but selects the stimulus emergence 0 to 400 ms for ERO analysis to explore the differences of theta and alpha oscillations under different conditions. In the occipital region, the time window is considered for analysis of LPP (400–900 ms), for theta and alpha. Although none of the ERO effects in the occipital region reached statistical significance, all corresponding results (including *F*, *p*, and *ηp*^2^ values for both theta and alpha oscillations) are fully reported the Supplemental Materials (Part 3). For clarity and conciseness, only the significant effects observed in the frontocentral region are described in the main text (Fig. 6).

**Theta**

A 2 (with/without sound) × 3 (picture type: negative, positive, neutral) repeated measures ANOVA is performed on theta oscillations (4–7 Hz, 0–400 ms) in frontocentral regions. The results show that the main effect of sound condition is significant (*F* (1,26) = 81.013, *p* < 0.001, *ηp*^2^ = 0.757), the main effect of picture type is significant (*F* (2,52) = 3.671, *p* = 0.032, *ηp*^2^ = 0.124), and the interaction of the two factors is not significant (*F* (2,52) = 2.875, *p* = 0.065, *ηp*^2^ = 0.100).

A simple effects analysis reveals significant differences in theta oscillations when sudden sound stimuli are applied across the three picture types (*F* (2,52) = 3.673, *p* = 0.032, *ηp*^2^ = 0.124). More precisely, the difference between the negative picture condition and the positive picture condition is significant (*p* = 0.011), the difference between the negative picture condition and the neutral picture condition is not significant (*p* = 0.066), and the difference between the positive picture condition and the neutral picture is not significant (*p* = 0.142). The negative picture condition has the largest theta oscillation energy (*M* = 3.09 ± 1.85), followed by the neutral picture condition (*M* = 2.82 ±1.75), and the positive picture condition (*M* = 2.79 ± 1.54). On the contrary, there is no significant difference in theta oscillation in the three picture types when no sound stimuli are applied (*F* (2,52) = 1.15, *p* = 0.324, *ηp*^2^ = 0.006).

**Alpha**

A 2 (with/without sound) × 3 (picture type: negative, positive, neutral) repeated measures ANOVA is performed on alpha oscillations (8–12 Hz, 0–400 ms) in frontocentral regions. The results show that the main effect of sound condition is significant (*F* (1,26) = 61.029, *p* < 0.001, *ηp*^2^ = 0.701), the main effect of picture type is significant (*F* (2,52) = 5.294, *p* = 0.008, *ηp*^2^ = 0.169), and the interaction of the two factors is significant (*F* (2,52) = 3.453, *p* = 0.039, *ηp*^2^ = 0.117).

A simple effects analyses reveals significant differences in alpha oscillation energy between the three emotional picture conditions when a sudden sound startle stimulus is applied (*F* (2,52) = 5.036, *p* = 0.010, *ηp*^2^ = 0.162). More precisely, the difference between the negative picture condition and the positive picture condition is significant (*p* = 0.013), the difference between the negative picture condition and the neutral picture condition is significant (*p* = 0.036), and the difference between the positive picture condition and the neutral picture is not significant (*p* = 0.766). The alpha oscillation energy is the greatest in the negative picture condition (*M* = 1.83 ± 1.25), followed by the positive picture condition (*M* = 1.57 ± 0.93), and finally the neutral picture condition (*M* = 1.54 ± 1.08). However, there is no significant difference in alpha oscillation energy between the three emotional picture conditions when no sudden sound stimuli are applied (*F* (2,52) = 0.283, *p* = 0.755, *ηp*^2^ = 0.011).

## Discussion

This study studies the influence of emotional factors on the startle reflex based on the EMS paradigm. The results of ERP analysis show that the Probe P3 and LPP components significantly differ across the three types of emotional picture conditions when a sudden startle stimulus is applied. On the other hand, the ERO analysis shows that the early theta and alpha oscillations in the occipital region of participants after the application of the sound stimuli, are greater in the negative picture condition than in the positive and neutral picture conditions. It is worth noting that, rather than introducing a novel neural indicator, the present study extends previous EMS-based EEG research by demonstrating that multiple neural indices can jointly characterize startle reflex modulation across different emotional conditions.

### Influence of emotional factors on startle reflex response

This study demonstrates that the startle reflex is strongly modulated by emotional context, with significant differences in attentional resource allocation across emotional conditions. The ERP results reveal that, in the absence of a startle stimulus, there are no significant differences in ERP amplitudes between the three emotional conditions at the midpoint of picture presentation, indicating that the emotional modulation was not evident at this stage, most likely due to baseline correction and the absence of the startle stimulus. However, when a startle probe is introduced, the attentional resources allocated to the sound vary by emotional condition, such that negative pictures elicited the greatest allocation of attentional resources, thereby leaving fewer available resources to process the startle stimulus, which was reflected in a smaller Probe P3 amplitude. These findings contrast with Leite et al. (2012), who reported the strongest inhibition of the Probe P3 amplitude in the positive condition. The discrepancy may be attributed to the specific emotional stimuli used in this study, which included only highly arousing negative, positive, and neutral images, possibly leading to heightened attention to negative stimuli (Solomon, DeCicco & Dennis, 2012). Importantly, the LPP findings in the present study support the notion that emotional factors continue to influence the startle reflex during later processing stages, as evidenced by the consistent patterns observed in LPP amplitude.

In the passive viewing of pictures, the study confirms the effectiveness of the emotional stimuli in eliciting corresponding emotional responses. The P3 and LPP components show significant differences across all emotional conditions, with the largest amplitudes in the negative picture condition. This complements the startle reflex findings, as the observed “complementary” pattern between passive viewing and post-stimulus ERP components highlights the role of motivated attention. Schupp et al. (1997) suggest that this pattern reflects the dynamic allocation of attentional resources, where the initial presentation of emotional pictures activates motivational systems, leading to enhanced attention during passive viewing. Subsequently, the shared resources between tasks result in reduced attentional allocation to the startle stimulus, as evidenced by lower Probe P3 amplitudes in the negative condition. The ERO analysis further supports these findings by revealing greater theta and alpha oscillations in the negative picture condition after the startle stimulus. Theta oscillations, closely linked to the P3 component, and alpha oscillations, indicative of frontocentral inhibition of emotional stimuli (Uusberg et al., 2013), underscore the differential neural processing of emotional stimuli.

Interestingly, previous psychophysiological studies have reported that subjective emotional arousal and physiological defensive responses are not always coupled (Mauss et al., 2005). In contrast, our findings showed a consistent enhancement across both subjective emotional experience and neural indices (LPP and oscillatory activity), suggesting that the relationship between emotional experience and physiological responses is not uniform across paradigms. This contrast emphasizes that the degree of alignment between emotional experience and physiological responses may vary depending on the stimulus characteristics and the measurement modality, highlighting the value of integrating multiple indicators when investigating affective processing.

Beyond differences in arousal, prior studies have emphasized that affective responses are also shaped by the semantic content and motivational relevance of emotional scenes. For instance, Bradley et al. (2001) demonstrated that defensive and appetitive motivational systems respond differently depending on the semantic meaning of affective pictures, even when arousal levels are comparable. Similarly, Cuthbert et al. (2000) found that ERP components such as the LPP are modulated by affective meaning rather than arousal alone. Further evidence from Bradley, Sapigao & Lang (2017) indicated that semantic features of emotional scenes influence physiological indices such as pupil dilation. In addition, De Cesarei & Codispoti (2011) reported that both LPP amplitude and alpha-band desynchronization are affected by picture semantics as well as emotion category. Together, these findings suggest that balancing arousal ratings alone may not ensure equivalent motivational relevance, and the semantic properties of emotional stimuli likely contributed to the neural differences observed in the present study.

### Joint ERP-oscillatory signatures of emotion-modulated startle processing

Beyond the interpretation of individual ERP components or oscillatory effects, the present findings contribute to a growing body of research emphasizing the importance of considering joint neural signatures when examining emotion-modulated startle processing. Recent EEG studies increasingly suggest that emotional modulation of defensive responding is not adequately captured by a single neural index, but rather emerges from coordinated changes across time-locked event-related potentials and non-phase-locked oscillatory dynamics (Keil et al., 2022; Codispoti, De Cesarei & Ferrari, 2023).

Within this framework, startle-elicited ERP components such as the Probe P3 are now commonly interpreted as reflecting context-dependent attentional allocation rather than a simple orienting response to acoustic stimulation. Contemporary EMS research has highlighted that probe-locked P3 modulation varies systematically with emotional context, task demands, and stimulus timing, underscoring its sensitivity to competitive processing between motivationally salient stimuli and secondary probes (Cuthbert et al., 2000; Schupp et al., 1997). Importantly, these effects are increasingly viewed as embedded within an ongoing stream of affective processing rather than isolated, transient responses.

Complementing ERP findings, low-frequency oscillatory activity has gained prominence as a critical index of emotion-related neural dynamics. Recent affective EEG literature consistently demonstrates that theta- and alpha-band activity track motivational relevance, attentional engagement, and defensive processing demands during emotional picture viewing (Güntekin & Başar, 2014). From this perspective, oscillatory modulation following startle probe onset may reflect sustained adjustments in cortical excitability and resource allocation that extend beyond the temporal resolution of ERP amplitudes alone. The convergence of ERP and oscillatory evidence therefore supports a multilevel account in which emotional context shapes both discrete event-related responses and broader neural state dynamics. Crucially, recent methodological discussions caution against overextending the functional interpretation of startle-related EEG effects without careful consideration of design constraints and analytic choices. Variability in probe timing, emotional stimulus selection, and sample characteristics has been shown to substantially influence both ERP and oscillatory outcomes in EMS paradigms (Codispoti, De Cesarei & Ferrari, 2023; Lim, Lew & Ang, 2024). As such, current consensus emphasizes mechanistic interpretation over application-oriented claims, particularly when external validation is limited or sample sizes are modest.

Taken together, the present results align with contemporary views that the EMS paradigm, when combined with EEG, is best understood as a tool for characterizing multistage emotional modulation of defensive processing rather than as a single-index measure of reflex magnitude. By demonstrating convergent modulation across ERP and oscillatory domains, this study contributes to a growing literature advocating for integrative neural approaches to understanding how emotional significance shapes defensive responding across multiple temporal and functional levels.

### Limitations

Despite the contributions of this study, several limitations must be acknowledged. First, the fixed timing of the startle stimulus at four seconds after picture presentation may have led to response patterns based on anticipation. However, drawing from Bradley, Codispoti & Lang (2006), where no significant differences in Probe P3 amplitude were observed between emotional conditions with variable timing, we cautiously speculate that this temporal fixation may not significantly influence the startle reflex. Nevertheless, future studies could adopt variable probe onset times to further minimize potential expectancy effects. Second, the relatively modest sample size may have limited the sensitivity to detect smaller effects, particularly in oscillatory measures. Although the present findings revealed consistent patterns across ERP and ERO indices, replication with larger samples would help to strengthen the robustness and generalizability of these effects. Third, while EEG provides valuable insights into the neural correlates of the startle reflex, the absence of EMG measures limits the ability to directly quantify the reflexive response. Future research integrating EEG and EMG techniques could offer a more comprehensive characterization of both central and peripheral components of the startle reflex.

## Conclusion

This study provides a comprehensive analysis of how emotional factors modulate the startle reflex, revealing that these effects begin with early attentional resource allocation and persist through later processing stages. The differential impact of emotional factors on the startle reflex was most clearly reflected in variations in theta and alpha oscillations, with stronger effects observed under negative emotional conditions.

## Supplemental Information

10.7717/peerj.21136/supp-1 Supplemental Information 1 Raw data.

10.7717/peerj.21136/supp-2 Supplemental Information 2 Supplementary Material. Part 1. The selection of IAPS pictures Part 2. The topographic map of Probe P3 and LPP Part 3. The ERO result in the occipital region Part 4. Participant state anxiety and trait anxiety scores Part 5. Confusion Matrix for Machine Learning

## Additional Information and Declarations

### Data Availability

The following information was supplied regarding data availability:

The raw data is available in the Supplemental File.
