# Supplementary material for: Neural mechanisms of emotion-modulated startle reflexes: insights from ERP and EEG oscillations
Source: PeerJ. 2026 Apr 21;14:e21136. doi: 10.7717/peerj.21136 (PMC13108465; doi:10.7717/peerj.21136)
Supplement: Supplemental Information 2 — Part 1. The selection of IAPS pictures Part 2. The topographic map of Probe P3 and LPP Part 3. The ERO result in the occipital region Part 4. Participant state anxiety and trait anxiety scores Part 5. Confusion Matrix for Machine Learning [file peerj-14-21136-s002.docx]

**Supplementary Material** for

**Neural mechanisms of emotion-modulated startle reflexes: Insights from ERP and EEG oscillations**

Fangfang Long^a,b^, Lulu Hou^c^ , Renlai Zhou^b, d*^

a. School of Psychology, Guizhou Normal University, Guiyang, 550025, China.

b. Department of Psychology, Nanjing University, Nanjing 210023, China.

c. School of Psychology, Shanghai Normal University, Shanghai 200233,China.

d. Department of Radiology, Nanjing Drum Tower Hospital, the Affiliated Hospital of Nanjing University Medical School, Nanjing, 210008, China.

* Corresponding author: Renlai Zhou

Professor, Department of Psychology, Nanjing University, Room 418, Heren Hall, 163 Xianlin Avenue, Nanjing, 210023, P.R. China

Tel: +86 13520001589; Fax: +86 25 89680960

1. mail address: [rlzhou@nju.edu.cn](mailto:rlzhou@nju.edu.cn).

**This Word file includes:**

Part 1.The selection of IAPS picture

Part 2.The topographic map of Probe P3 and LPP

Part 3. The ERO result in the occipital region

Part 4. Description of Variables in the *raw_data.xlsx* File

**Part 1.The selection of IAPS picture**

In the IAPS picture, the negative picture selected is as follows:

1019、1030、1040、1050、1052、1080、1111、1113、1220、1280、1300、1310、1945、2053、2120、2800、3000、3015、3030、3051、3053、3060、3061、3062、3064、3071、3080、3100、3102、3120、3130、3140、3150、3160、3168、3170、3250、3261、3266、3350、3400、3530、3550、6260、6300、6313、6550、6570、6831、8230、9042、9140、9320、9405、9420、9433、9570、9921、1051、1090、1120、3063、6250_1、9250、9400、9430、9571、9600、9490、9253、6570_1、1070、3010、6510、1201、1321、3550、9920、1930、6560；

The neutral picture selected is as follows:

1340、1601、1604、2050、2000、1999、2501、2550、2530、2345、2331、2311、2070、2030、1710、1750、2091、2080、5200、5220、5760、5780、5731、8034、8162、8330、8350、8380、8461、8496、8497、8540、1440、2040、2058、2150、2208、2216、2303、2340、2655、2352、2391、5001、5201、5600、5830、5831、5910、7270、7282、7283、7351、7390、7475、8190、4571、5849、7325、8032、8470、8500、8531、2341、4617、5300、5849、1463、8120、5480、2900、8502、4641、8501、4700、8031、8185、8503、8030、8210；

The positive picture selected is as follows:

2200、2206、2280、2372、2485、2490、2495、2518、2520、2570、2575、2590、2650、2749、2850、3300、5130、5531、5532、6000、6150、7002、7004、7009、7010、7020、7025、7030、7031、7034、7035、7040、7050、7060、7080、7095、7096、7100、7130、7150、7170、7180、7185、7224、7234、7235、7490、7550、7590、7595、7700、7820、7950、7190、7233、7705、7090、7140、7175、7006、7000、7217、7211、7187、7545、5030、2514、2480、7183、2221、7500、9090、2383、2312、2890、7491、7710、7237、7034、5890

**Part 2.The topographic map of Probe P3 and LPP**

Topographical maps of the brain in three different types of emotional picture conditions when sudden sound stimuli were applied have been presented in the manuscript. In this section, we add the topography of the participants' brains in the other half of the trials in which no sound stimuli were presented.


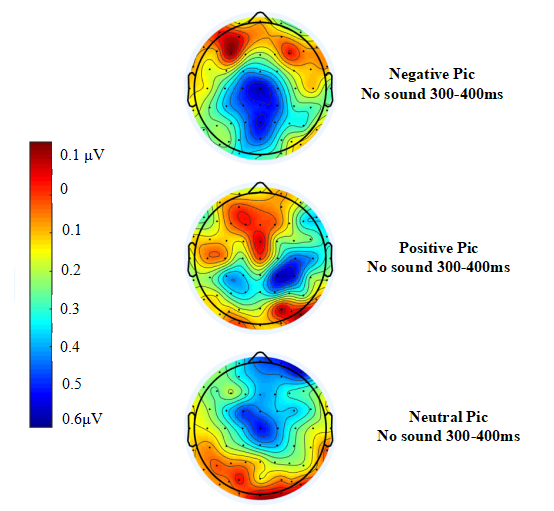


Figure1. Topographic maps for 300-400ms in three emotion picture conditions when no sound stimuli were applied.


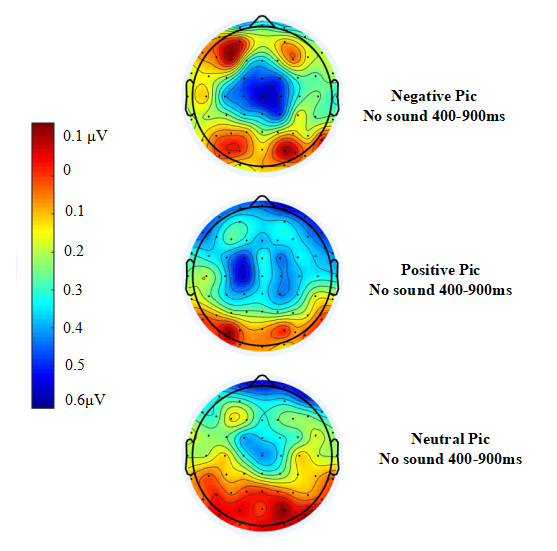


Figure2. Topographic maps of 400-900ms in three mood picture conditions when no sound stimuli were present.

**Part 3. The ERO result in the occipital region**

**theta**

A 2 (with/without sound) x 3 (picture type: negative, positive, neutral) repeated measures ANOVA was performed for theta oscillations (4-7 Hz, 400-900 ms) in the occipital region. The results showed that the main effect of sound condition was not significant(*F*(1,26) = 0.002, *p* = .968,*ηp*^2^ < 0.001), the main effect of picture type was not significant (*F*(2,52) = 2.454, *p* = .096,*ηp*^2^ = 0.086), and the two-factor interaction was not significant (*F*(2,52) =0.981, *p* = .382,*ηp*^2^ = 0.036).

**alpha**

A 2 (with/without sound) x 3 (picture type: negative, positive, neutral) repeated measures ANOVA was performed for alpha oscillations (8-12 Hz, 400-900 ms) in the occipital region. The results showed that the main effect of sound condition was not significant (*F*(1,26) = 1.601, *p*= .217,*ηp*^2^ = 0.058), the main effect of picture type was not significant (*F*(2,52) =1.654, *p* = .201,*ηp*^2^ = 0.060), and the two-factor interaction was not significant (*F*(2,52) = 0.917, *p*= .406,*ηp*^2^ = 0.034).

**Part 4. Description of Variables in the *raw_data.xlsx* File**

The file raw_data.xlsx contains two sheets summarizing the electrophysiological data used in the present study.

Sheet 1 contains the P3 and LPP amplitudes measured during passive picture viewing across emotional conditions (negative, positive, and neutral).

Sheet 2 presents the ERP and ERO results from the acoustic startle phase (the fourth second of picture presentation), including the P3 and LPP amplitudes under both sound and no-sound conditions, as well as the corresponding theta and alpha power values extracted from the frontocentral and occipital regions, respectively.
